# Advances in Designing and Developing Vaccines, Drugs, and Therapies to Counter Ebola Virus

**DOI:** 10.3389/fimmu.2018.01803

**Published:** 2018-08-10

**Authors:** Kuldeep Dhama, Kumaragurubaran Karthik, Rekha Khandia, Sandip Chakraborty, Ashok Munjal, Shyma K. Latheef, Deepak Kumar, Muthannan Andavar Ramakrishnan, Yashpal Singh Malik, Rajendra Singh, Satya Veer Singh Malik, Raj Kumar Singh, Wanpen Chaicumpa

**Affiliations:** ^1^Division of Pathology, ICAR-Indian Veterinary Research Institute, Bareilly, India; ^2^Central University Laboratory, Tamil Nadu Veterinary and Animal Sciences University, Chennai, India; ^3^Department of Biochemistry and Genetics, Barkatullah University, Bhopal, India; ^4^Department of Veterinary Microbiology, College of Veterinary Sciences and Animal Husbandry, Agartala, India; ^5^Immunology Section, ICAR-Indian Veterinary Research Institute, Bareilly, India; ^6^Division of Veterinary Biotechnology, ICAR-Indian Veterinary Research Institute, Bareilly, India; ^7^Division of Virology, ICAR-Indian Veterinary Research Institute, Nainital, Uttarakhand, India; ^8^Division of Biological Standardization, ICAR-Indian Veterinary Research Institute, Bareilly, India; ^9^Division of Veterinary Public Health, ICAR-Indian Veterinary Research Institute, Bareilly, India; ^10^ICAR-Indian Veterinary Research Institute, Bareilly, Uttar Pradesh, India; ^11^Center of Research Excellence on Therapeutic Proteins and Antibody Engineering, Department of Parasitology, Faculty of Medicine SIriraj Hospital, Mahidol University, Bangkok, Thailand

**Keywords:** Ebola virus, Ebola virus disease, vaccines, prophylactics, drugs, therapeutics, treatment

## Abstract

Ebola virus (EBOV), a member of the family *Filoviridae*, is responsible for causing Ebola virus disease (EVD) (formerly named Ebola hemorrhagic fever). This is a severe, often fatal illness with mortality rates varying from 50 to 90% in humans. Although the virus and associated disease has been recognized since 1976, it was only when the recent outbreak of EBOV in 2014–2016 highlighted the danger and global impact of this virus, necessitating the need for coming up with the effective vaccines and drugs to counter its pandemic threat. Albeit no commercial vaccine is available so far against EBOV, a few vaccine candidates are under evaluation and clinical trials to assess their prophylactic efficacy. These include recombinant viral vector (recombinant vesicular stomatitis virus vector, chimpanzee adenovirus type 3-vector, and modified vaccinia Ankara virus), Ebola virus-like particles, virus-like replicon particles, DNA, and plant-based vaccines. Due to improvement in the field of genomics and proteomics, epitope-targeted vaccines have gained top priority. Correspondingly, several therapies have also been developed, including immunoglobulins against specific viral structures small cell-penetrating antibody fragments that target intracellular EBOV proteins. Small interfering RNAs and oligomer-mediated inhibition have also been verified for EVD treatment. Other treatment options include viral entry inhibitors, transfusion of convalescent blood/serum, neutralizing antibodies, and gene expression inhibitors. Repurposed drugs, which have proven safety profiles, can be adapted after high-throughput screening for efficacy and potency for EVD treatment. Herbal and other natural products are also being explored for EVD treatment. Further studies to better understand the pathogenesis and antigenic structures of the virus can help in developing an effective vaccine and identifying appropriate antiviral targets. This review presents the recent advances in designing and developing vaccines, drugs, and therapies to counter the EBOV threat.

## Introduction

Ebola virus (EBOV; *Zaire ebolavirus*) is the causative agent of a severe hemorrhagic fever disease, Ebola virus disease (EVD; formerly called Ebola hemorrhagic fever). It was first recognized in 1976 in northern Democratic Republic of Congo, at that time Zaire (1–3). Since then, EVD is endemic in Africa. Fruit bats are the best-known reservoirs of EBOV (4). EVD is a well-established zoonotic disease; the initial cases of the EVD outbreaks occur after contact with reservoir or materials contaminated with the virus and followed by human-to-human transmission (5). EBOV is not only a serious public health issue but now also designated as category A pathogen and considered as a potential bioterrorism agent (6, 7). EBOV causes high mortality rates of up to 88% in the infected humans (8); therefore, it is classified as a risk group 4 agent and handled under biosafety level-4 containment. The risk of mortality is relatively greater in the elderly and/or patients with high viral load and poor immune response at the initial stage of the infection (9).

The EBOV belongs to the *Filoviridae* family and has a unique thin filamentous structure that is 80-nm wide and up to 14-µm long. Its envelope is decorated with spikes of trimeric glycoprotein (GP1,2) which are responsible for mediating viral entry into target cells (function of GP1) (10) and release of viral ribonucleoprotein from endosome to cytoplasm for replication (function of GP2) (11, 12). EBOV infects primarily humans, simians, and bats; but other species such as mice, shrew, and duikers may also contact infection (3, 13). Of the five identified EBOV species, four species, *viz*., EBOV, Sudan virus (SUDV; *Sudan ebolavirus*), Tai Forest virus (TAFV; *Tai Forest ebolavirus*, formerly *Côte d’Ivoire ebolavirus*), and Bundibugyo virus (BDBV; *Bundibugyo ebolavirus*), are known to infect humans and cause disease, whereas Reston virus (RESTV; *Reston ebolavirus)* is non-human primate (NHP) pathogen.

After an initial incubation period of 3–21 days, the disease progresses quickly to fever, intense fatigue, diarrhea, anorexia, abdominal pain, hiccups, myalgia, vomiting, confusion, and conjunctivitis (14) which may lead to the loss of vision (15). EBOV can spread from males to females through semen (16) and from mother to fetus and infant during gestation and lactation, respectively (17). Of the note, in an EBOV-infected patient, higher concentration of Ebola viral RNA in semen was noticed during the recovery period than the viral concentration in the blood during peak time of infection, suggesting male genital organ as virus predilection site for replication (18). Usually the human immune system mounts a response against infectious pathogens by sensing the pathogen-associated molecular patterns *via* a variety of pathogen-recognition receptors. Nevertheless, in the case of EBOV, innate immunity is impaired by the immunosuppressive viral proteins including VP35 and VP24, and lymphocytes are depleted as a result of apoptosis caused by inappropriate dendritic cell (DC)–T-cell interaction (7, 19). A thorough understanding on the pathogenesis of this deadly virus is essential because of its severe health impacts (20).

The increased incidences and fast spread of EBOV paving into a pandemic flight has compelled more focus of research to develop strategies and remedial measures for mitigating the impact and consequential severity of the viral infection. Even before delineating the less studied Ebola viral genome fully, researchers throughout the globe and health industry were pressured to focus on the development of effective and safe Ebola vaccines and therapeutics (21, 22). As of now, no licensed vaccines and direct-acting anti-EBOV agents are available to protect against the lethal viral infection or to treat the disease. To minimize the suffering, EBOV-infected patients are only provided with symptomatic treatment and supportive care. Because of its high pathogenicity and mortality rate, preventive measures, prophylactics, and therapeutics are essential, and researchers worldwide are working to develop effective vaccines, drug, and therapeutics, including passive immunization and antibody-based treatments for EVD (23–26). Prior to the 2014–2016 EBOV outbreak in West Africa, which has been the deadliest EBOV outbreak to date, convalescent blood products from survivors of EVD represented the only recommended treatment option for newly infected persons. Administration of monoclonal antibody (mAb) cocktails (ZMapp, ZMAb, and MB-003) as post-exposure prophylactics have been found to reverse the advanced EVD in NHPs and/or effectively prevented morbidity and mortality in NHPs (27–30).

There is the need for an effective vaccine against EBOV, especially in high-risk areas, to prevent infections in physicians, nurses, and other health-care workers who come into contact with diseased patients (31). Regular monitoring and surveillance of EBOV is essential to control this disease. In the EBOV outbreak, novel surveillance approaches include contact tracing with coordination at the national level and “lockdown” periods, during which household door-to-door reviews are conducted to limit the spread of the virus. Swift identification and confirmation of the Ebola cases and immediate follow-up of appropriate prevention and control measures, including safe burial of dead persons, are crucial practices to counter EBOV (32).

After the onset of EVD, treatment is required, whereas, when EBOV is circulating in population dense areas before infection, prophylactic measures like vaccination are necessary. One of the main challenges in containing EBOV is its presence in remote areas that lack technology and equipment to limit the virus spread. Because of its lethality, EBOV can only be handled in laboratories with biosecurity level-4 containment; thus, only few laboratories in the world can conduct EBOV research and testing of the counter measures against the authentic virus. Recent efforts by several organizations have focused on identifying effective therapies and developing appropriate vaccination strategies (33). Several drugs and vaccines have been developed against EBOV, and the production of low-cost drugs and vaccines against EBOV is essential for everyone, including those in the high-risk areas of the world, to be protected (26, 34). As of the acquisition of better knowledge against the pathogen due to improvement in the field of genomics and proteomics, there has been expansion in the field of vaccine synthesis where epitope-based vaccines are gaining top priority (35–37).

The present review aims to discuss advances in designing and development of EBOV vaccines, drugs, antibody-based treatments, and therapeutics, and their clinical efficacy in limiting EVD, thereby providing protection against the disease and alleviating high public health concerns associated with EBOV.

## Advances in Developing Vaccines Against EBOV

There is a clear need for an effective vaccine to prevent the rapid spread of EVD. An inactivated EBOV vaccine was first produced in 1980. This vaccine was tested for efficacy in guinea pigs (7). Since that time, several vaccines against EBOV have been developed, but no vaccine is licensed and available in the market (7). After the massive 2014–2016 outbreak of EBOV, several researchers have begun working to develop an effective vaccine (38). For an EBOV vaccine candidate, a long-lasting immune response is essential; as EBOV remains in the seminal fluid of EVD survivors as long as 401 days post-infection (39, 40). Keeping this window of virus persistence, a vaccine conferring immunity at least for 2 years is recommended by the Wellcome Trust-CIDRAP Ebola Vaccine Team B initiative (41). Vaccines like the chimpanzee adenovirus type 3 (ChAd3)-based non-replicating ChAd3-EBO vaccine, prime-boost recombinant adenovirus type 26 vector (Ad26.ZEBOV) followed by the modified vaccinia Ankara vector (MVA-BN-Filoa) vaccine, adenovirus 5-vectored EBOV vaccine, EBOV DNA vaccine, and recombinant vesicular stomatitis virus (rVSV) vector-based vaccine are undergoing clinical trials to evaluate their efficacy against EVD (38). The RNA-dependent RNA polymerase (L) epitope-based vaccine was designed using immunoinformatics. Various software have been used to analyze immunological parameters, and this epitope vaccine was found to be a good candidate for use against EVD (42). Two conserved peptides of EBOV, 79VPSATKRWGFRSGVPP94 from GP1 and 515LHYWTTQDEGAAIGLA530 from GP2, were identified as targets for the development of an epitope-based vaccine (43). Collection of the sequences of EBOV glycoproteins and examination for determining the proteins with greatest immunogenicity have been performed using *in silico* methods. The best corresponding B and T cell epitopes included peptide regions encompassing residues 186–220 and 154HKEGAFFLY162, respectively. Such predicted epitopes can confer the long-lasting immunity against EBOV with better ability of protection (36).

Ebola virus-GP fused with the Fc fragment of a human IgG1 subunit vaccine administrated with alum, QS-21, or polyinosinic-polycytidylic acid-poly-l-lysine carboxymethylcellulose adjuvant induced strong humoral immunity in guinea pigs (44). Effectiveness of a ring vaccine using rVSVΔG/EBOVGP in cases of simulated EBOV disease was studied and even this approach can be employed during an outbreak situation (45). Notably, the neutralizing antibodies play a major role in conferring protection against EBOV infections. Thus, an EBOV vaccine capable of effectively inducing a long-lasting neutralizing antibody response is desirable for developing appropriate prevention strategies in combating the infection. In this line, the mucin-like domain of EBOV envelope glycoprotein GP1 has been identified to be critical in induction of protective humoral immune response (46, 47). Filorab 1 vaccine revealed desirable immunogenicity without the side effects. The main advantage of this vaccine is its higher immune response induction in chimpanzees (captive) when given orally and also with a single dose [instead of multiple doses as is required by virus-like particle (VLP) vaccine] (48). Modified mRNA-based vaccine constructs, formulated with lipid nanoparticles (LNPs) to facilitate delivery, are being tested against EBOV challenge in guinea pigs. These mRNAs induced robust immune responses and conferred up to 100% protection from the infection (49).

It is important to note that compilation of data in relation to immune responses (both induced by vaccines and natural infection) and the records of community members showing IgG seropositivity should be kept systematically. Assimilation of such information will help to handle next outbreaks with more rigidity, thereby helping to check EVD-associated disasters at an early stage (50). *Vis-à-vis* public health workers should also be vaccinated and mass vaccination programs should be undertaken through standardized and coordinated efforts (51).

The following section describes the various types of vaccines and vaccine platforms which are being explored for the development of a successful EBOV vaccine.

### Inactivated Vaccines

Even though inactivated vaccines suffer with the problem of reversion to virulence due to inadequate viral inactivation, various strategies have been constantly explored in developing safe and potent non-replicating vaccine candidates for combating the EBOV infection (52). Both heat- and formalin-inactivated EBOV have been found protective against EBOV infection in a guinea pig model. Inclusion of inactivated vaccine with EBOV E-178 along with interferon (IFN) and immune plasma saved the life of a scientist working on EBOV (53). The protective efficacy of liposome-encapsulated irradiated EBOV, tested in a mouse model, was 100%. However, these viral particles failed to protect NHPs (54). This suggests that murine model is excellent for evaluating vaccine efficacy, but the level of protection might be different in different species and, hence, it is essential to test vaccines in NHPs before proceeding to clinical trials in humans. Heat-, formalin-, or gamma irradiation-killed EBOV vaccines have been found ineffective against EBOV disease; thus, the novel effective vaccine is essentially required (55).

### DNA Vaccines

In DNA vaccines, plasmids are used to express immunogenic antigens. This is an attractive vaccine approach because of the ease of production and simplicity. In addition, DNA vaccine induces both humoral and cellular immune responses. A three-plasmid DNA vaccine comprising the transmembrane-deleted GP sequences from EBOV species Zaire and SUDV-Gulu as well as nucleoprotein (NP) sequence from EBOV was tested in healthy adults. The vaccine was well tolerated, and both CD4^+^ and CD8^+^ T cell responses were elicited (56). An EBOV GP DNA vaccine designed on a consensus alignment of GPs (from strains obtained during 1976–2014), delivered intramuscularly and then electroporated, elicited a strong T cell response, and protected 100% of experimental mice from lethal challenge with EBOV (57). The DNA from three strains of EBOV was used to prime human volunteers and boosted with attenuated adenovirus, which acted as delivery vehicle for EBOV DNA into antigen-presenting cells, induced significant humoral- and cell-mediated immune (CMI) responses (58). Intramuscular inoculation of the DNA vaccine through electroporation with DNA plasmid containing codon-optimized GP genes of EBOV elicited high levels of IgG and a strong CMI response (measured by IFN-γ ELISpot assay) in cynomolgus macaques (59).

Though the preliminary trials using DNA constructs have provided the acceptable safety profiles, the development of low immune titer for a shorter window necessitates repeated vaccinations to overcome this problem. Thus, the use of a potent vaccination regimen based on DNA vaccine platforms does not appear logical for a large population (60, 61).

### Virus-Like Particles

Ebola VLPs (EBOV-VLPs or eVLPs) are generated from the expression of viral transmembrane glycoprotein (GP) and structural matrix protein (VP40) in mammalian cells, which undergo self-assembling and budding from host cells and display morphological similarity to infectious EBOV particles (47). Baculovirus-derived eVLPs comprising GP, VP40, and NP of EBOV have been found to induce human myeloid DC maturation, suggesting their immunogenicity. Baculovirus-generated VLPs were able to elicit similar levels of protection as 293T cell-derived VLPs and showed protection against virus challenge in a dose-dependent manner (62). Nano-VLPs, produced by sonication of VLPs and filtering to have a mean diameter of approximately 230 nm, increased their thermostability. Unlike native VLPs where GP protein is denatured in a solution by heating, the nano-VLP maintained the conformational integrity of the GP protein at temperature up to 70°C and could confer protection in a mouse model (63). VLP containing only VP40 was sufficient to protect mice from EBOV infection. VLP injection leads to an enhanced number of natural killer (NK) cells, which play a crucial role in innate immune protection against lethal EBOV. NK cell protection is dependent on perforin, but not recombinant viral vector vaccines on IFN-gamma secretion (64).

Ebola virus VP40 and GP have been demonstrated to interact with the host protein, BST2, and are associated with viral infections by trapping the newly assembled enveloped virions at the plasma membrane in the infected cells, ultimately induce NF-κB activity. The effects of EBOV GP1,2, VP40, and BST2 converge on an intracellular signaling pathway leads to neddylation, resulting in the additive response with respect to the induction of NF-κB activity. Exploring the dynamics of this interaction could provide targets for vaccine developments and therapies that can modulate the inflammatory response during EVD (65).

Quantitation of EBOV antigenic particles using proteomic assays like liquid chromatography high resolution mass spectrometry method can be employed for determining the batch quality of vaccine constructs as well as in optimizing the dosages by assessing the amount of GP1 needed to confer effective protection (47).

It is to be noted that though anti-EBOV antibody can mediate effective protection, VLP-vaccinated murine models were shown to survive the EBOV challenge in the absence of detectable serum anti-EBOV antibodies (66). It could also be revealed that adjuvant signaling may circumvent the necessity for B-cell immunity in conferring protection against EBOV. These studies can be valuable for the future characterization, development, and optimization of effective EBOV vaccine candidates (66).

### Virus-Like Replicon Particles (VRPs)

The VRPs are the alternative to live-attenuated vaccines. The use of VRPs eliminates the risk of reversion to the original pathogenic form of live vaccine strains. To generate VRPs, generally filoviruses or alphaviruses are required. Here, while keeping the genes essential for replication, viral structural genes are deleted from full-length genomic cDNA clones. Viral structural genes are replaced with alternative gene(s) coding for an immunogen. Such replicons are able to replicate and transcribe upon transfection in competent cells. The resulting VRPs are able to infect cells only for one cycle. Because of the lack of structural genes, viral progeny are not formed. Viruses such as Venezuelan equine encephalitis virus (VEEV) can be used for production of EBOV antigen instead of structural proteins for the replicon vector. Thus, such vaccines are also quite safe (67). The gene inserted is typically GP, the main target of neutralizing antibodies. VRPs expressing EBOV VP24, VP30, VP35, and VP40 have been evaluated for their protective efficacy in a mouse model, but these were found not to be as protective as EBOV GP and NP antigens. VEEV replicons containing GPs from both EBOV and SUDV showed promising results in cynomolgus macaques after administration of a single dose. Here, two VRPs were constructed that contained the GP of EBOV or SUDV. The animals intramuscularly injected with both of the VRPs, survived viral challenge without exhibiting any clinical signs. The final results indicated that VRP-EBOV GP was able to confer cross-protection against SUDV, whereas VRP-SUDV GP was unable to provide complete protection against EBOV-Zaire challenge (68).

Recently, Ren et al. (69) constructed an alphavirus Semliki forest virus based recombinant replicon vector DREP for efficient and unchecked *ex vivo* co-expression of EBOV GP and VP40. Active immunization with recombinant DREP vectors possessing GP and VP40 induced cellular and humoral immune responses in murine model against EBOV antigens. This path breaking approach may provide key insights and strategies for designing further effective vaccines to contain EBOV permanently.

### Reverse Genetics System for EBOV Vaccine

A full-length recombinant EBOV infectious clone was constructed using cDNA. By employing reverse genetics method, viable but replication incompetent virus lacking entire VP30 ORF was constructed. The resultant EbolaΔVP30 is biologically contained and replication deficient, until VP30 is provided extraneously. Virus replication in cell culture was allowed by growing the virus in Vero cell line that stably expresses VP30, designated VeroVP30 (70). The safety of EbolaΔVP30 has been evaluated in mice and guinea pig model and was able to protect from lethal infection (71). The EbolaΔVP30 virus inactivated by using hydrogen peroxide protected NHPs after a single immunization. To avoid any incidence of potential recombination events that might result in regaining the replicative efficiency, the vaccine candidate was inactivated by hydrogen peroxide, that creates nicks and breakages in single- or double-stranded DNA or RNA and the virus is completely inactivated while retaining antigenic determinants unaffected (72).

### Recombinant Viral Vector Vaccines

Engineered viruses are gaining popularity because of their ability to efficiently induce CMI responses (a major part of adaptive immunity along with humoral response), as the antigen is expressed and processed in the cytoplasm. Replication-competent rVSV and chimpanzee adenovirus 3 (ChAd-3/cAd3) are the most efficient platforms for designing new vaccines (73). A recombinant vesiculovirus vector containing EBOV GP region (rVSVΔG/EBOVGP) was found to be highly effective after a single injection in NHPs (74, 75). The vaccine evaluated in pigs showed no disease development and no viral shedding. This indicated that the vaccine could be utilized for herd immunization and it also suggested the safety of the live-attenuated rVSVΔG/EBOVGP vaccine (76). Recently, this rVSVΔG/EBOVGP vaccine was evaluated in a randomized double blinded placebo phase III trial in 1,197 humans. There were no adverse effects or death following vaccination, supporting its use as a vaccine (77). The vaccine protected immunocompromised rhesus macaques that had a high number of CD4^+^ T cells (78). The rVSVΔG/EBOVGP vaccine was also studied for its efficacy as a therapy in rhesus monkeys after exposure to EBOV-Makona. This vaccine showed minimal prophylactic efficacy after exposure (79). Efficacy trials initiated to test the rVSV-vectored EBOV vaccine showed greater efficiency at the time of EVD outbreak, if deployed following the strategy of ring vaccination (80).

Another recombinant vaccine (VSV based), i.e., rVSV-Zaire EBOV has been shown to provide substantial protection. From 10th day of vaccination with this vaccine, no report of any disease was documented, which proved efficacy and effectiveness of rVSV-vectored vaccine in preventing EVD (81). It is interesting to note that seroconversion has been noticed in recipients of recombinant VSV-EBOV (rVSV-EBOV) vaccine by the end of fourth week (i.e., by 28 days) against the Kikwit strain glycoprotein (82). Another recombinant vaccine *viz*., rVSV-EBOV vaccine was tested as a candidate vaccine. This particular vaccine is under trial in human (phase II/III). It provides protection against only EBOV and is clinically efficient in the clinical set up of ring vaccination format (38, 83, 84). EBOV and SUDV glycoproteins have been assimilated into a cAdVax vector (adenovirus-based vaccine). In mice, this vaccine has provided full protection (85, 86). During recent outbreak in Democratic Republic of the Congo (DRC), rVSVΔG-EBOV-GP is being used for ring vaccination in the affected area. Though the vaccine is yet not approved and still under investigation.

In Russia, clinical trial of a vaccine, GamEvac-Combi, has been performed and has been approved to enter in phase III clinical trial (87). The vaccine GamEvac-Combi contained two heterologous expression systems. One is live-attenuated rVSV and the second is a recombinant replication-defective adenovirus type-5 (Ad5). Both the vectors are expressing the same glycoprotein. The rationale to use a combination of two vectors expressing glycoprotein of EBOV is that widely present preexisting immunity to Ad5 limits the use of Ad5 and also a negative correlation between EBOV glycoprotein-specific immune response and preexisting antibodies to Ad5 has been reported (88). Hence, prime immunization with VSV vectored vaccine and then boosting with AD5 vectored vaccine might contribute in compensating negative impacts of preexisting immune response to Ad5. This heterologous vaccine evoked glycoprotein-specific immune response in 100% volunteers on day 28th. Also, the vaccine is well tolerated and did not significantly altered the body physiological parameters and vital organs. In Liberia, Sierra Leone, and Guinea; the VSV and ChAd3 vectored vaccine are in focus (89).

Another study in mice models has reported that the adoption of a heterologous prime-boost vaccine strategy can result in a durable EBOV-neutralizing antibody response. The chimpanzee serotype 7 adenovirus vectors expressing EBOV GP (AdC7-GP) was used for priming and a truncated version of EBOV GP1 protein (GP1t) was used for boosting. Vaccination response studies showed that AdC7-GP prime/GP1t boost strategy was more potent in generating a sustained and strong immune response as compared to using an individual vaccine construct (90).

Replication-defective recombinant chimpanzee adenovirus type 3-vectored EBOV vaccine (cAd3-EBO) elicited both cell-mediated and humoral immunity in NHPs. A vaccine dose of 2 × 10^11^ particle units was found sufficient to induce protective immunity in the NHPs and to eliminate the effect of prior immunity to cAd3 (91). Recombinant VSV vaccine expressing EBOV GP and A/Hanoi/30408/2005 H5N1 hemagglutinin (VSVΔG-HA-ZGP) protected mice against challenge with both viruses and also cross-protected against H5N1 viruses (92).

The utility of adenovirus-vectored EBOV vaccines is limited with preexisting anti-adenoviral antibodies, which significantly lower the GP-specific humoral and T cell responses (88). Six mutations in the genome of MVA virus restrict its host specificity and make it unable to replicate in mammalian cells. A randomized study of a multivalent MVA vaccine encoding GPs from EBOV, SUDV, Marburg virus (MARV), and TAFV NP (MVA-BN-Filo) conducted in 87 participants resulted in no fever. The quadrivalent vaccine formulation has demonstrated the boosting up of both cellular and humoral immune responses against EBOV to several folds (93). Twenty-eight days after immunization, GP-specific IgG was detected with EBOV-specific T cell responses (94). EBOV GP and TAFV NP expressed in an MVA platform assembles into VLPs. Heterologous NPs enhanced VLP formation and offered GP-specific IgG1/IgG2a ratios comparable to those of MVA-BN-Filo (95).

Recombinant cytomegalovirus expressing EBOV GP was found to evoke protective immunity in rhesus monkeys challenged with EBOV (79). Baculovirus-expressed EBOV-Makona strain GP administered with Matrix-M (saponin adjuvant) showed better immunogenicity. Administration of Matrix M-adjuvanted vaccine resulted in increased IgG production and CD4^+^ and CD8^+^ T cell production (96). A human parainfluenza virus type 3-vectored vaccine expressing the GP of EBOV (HPIV3/EboGP) was developed as an aerosolized vaccine, and studies in Rhesus macaques showed 100% protection against challenge with EBOV (97).

Adenovirus 26 vectored glycoprotein/MVA-BN vaccine has recently passed the phase I trial (94). In the European countries including United Kingdom and United States, for the purpose of clinical trial, administration of ChAd-3 vectored vaccine has been adopted. This vaccine expresses the EBOV GP and is available in monovalent and divalent forms (91, 98).

Ebola vaccine potency trials employing replication defective adenoviral vectors (rAd) encoding EBOV GP have come up with promising results in NHP models. Based on such studies, multiple mutant glycoproteins were developed (such as glycoprotein with deleted transmembrane domain) which offers reduced *in vitro* cytopathogenicity but possessed reduced vaccine-mediated protection. In contrast to this, a point mutated glycoprotein has been reported to offer minimal cytopathogenicity and appropriate immune protection even with a two logs lower vaccine dose (99).

### Plant-Based Vaccines and Antibodies

Viral antigens, including GP, VP40, and NP, elicit protective immune responses. ZMapp, the cocktail of antibodies being used to treat EBOV, is a biopharmaceutical drug. To note, the component antibodies in ZMapp are manufactured in *Nicotiana benthamiana* using a rapid antibody manufacturing platform. Gene transfer is mediated by a viral vector, and the expression is transient. *N. benthamiana*-derived antibodies produced stronger antibody-dependent cellular cytotoxicity than the analogous anti-EBOV mAbs produced in a mammalian Chinese hamster kidney cell line (100). Phoolcharoen (101) expressed a GP1 chimera with the heavy chain of 6D8 mAb, forming an immune complex that was co-expressed with the light chain of the same mAb in leaves of tobacco plant. The ammonium sulfate-precipitated purified antibodies, along with poly(I:C) adjuvant, a synthetic analog of double-stranded RNA capable of interacting with toll-like receptor (TLR)-3, was found to elicit strong neutralizing anti-EBOV IgG. In addition, the immune complex along with poly(I:C) adjuvant was capable of stimulating a Th1/Th2 response. The experiment suggested the potential application of plant-produced Ebola immune complexes as vaccine candidates. EBOV VP40 was expressed in tobacco plants, and a mouse immunization study showed results that suggested this approach can be used to produce an EBOV vaccine (102).

The utility of plants as bioreactors for the bulk production of ZMapp could be considered to meet the required demand. The glycosylation pattern of mAbs may alter their efficiency and bioactivity, including their binding with the antigenic epitope. Several glycoforms of EBOV mAb13F6 have been prepared using a magnICON expression system. These glycoforms have human-like biantennary N-glycans with terminal N-acetylglucosamine, resulting in a structure similar to that of human mAbs. Hence, these are beneficial for humans (103).

Both RNA and DNA viruses have been modified to serve as plant-based vectors for the expression of heterologous proteins. Bean yellow dwarf virus, a single stranded-DNA virus, can replicate inside the nucleus of plant cells using their cellular machinery. A vector containing deletions in the coat-encoding genes and gene for the desired antigen may be inserted to form an expression cassette. The delivery of vectors to plants is *Agrobacterium*-mediated (23). mAbs against EBOV are produced by the process of agroinfiltration. In this context, it is noteworthy that lettuce acts as a very good host for the process of agroinfiltration. In lettuce cells, *Agrobacterium tumefaciens* has been used for delivering viral vectors (104). Neutralizing and protective mAb6D8 against EBOV has been expressed at a concentration of 0.5 mg/g of leaf mass. This quantity is similar to that generated in magnICON expression system (105). The plant-derived approach to vaccine development is attractive because of the large amount of transient proteins that can be expressed, with the potential for use during high demand for therapeutics and prophylactics (106). Advances in the field of vector expression like plant transient expression system and associated host cell engineering and manufacturing processes paved way for developing biopharmaceutical proteins and therapeutics in commercial basis. The great potentials of such novel approaches have been exploited for evolving therapeutics to counter emerging pandemics of EBOV and influenza that is evidenced from the production of experimental ZMapp antibodies (107).

**Figure 1:**
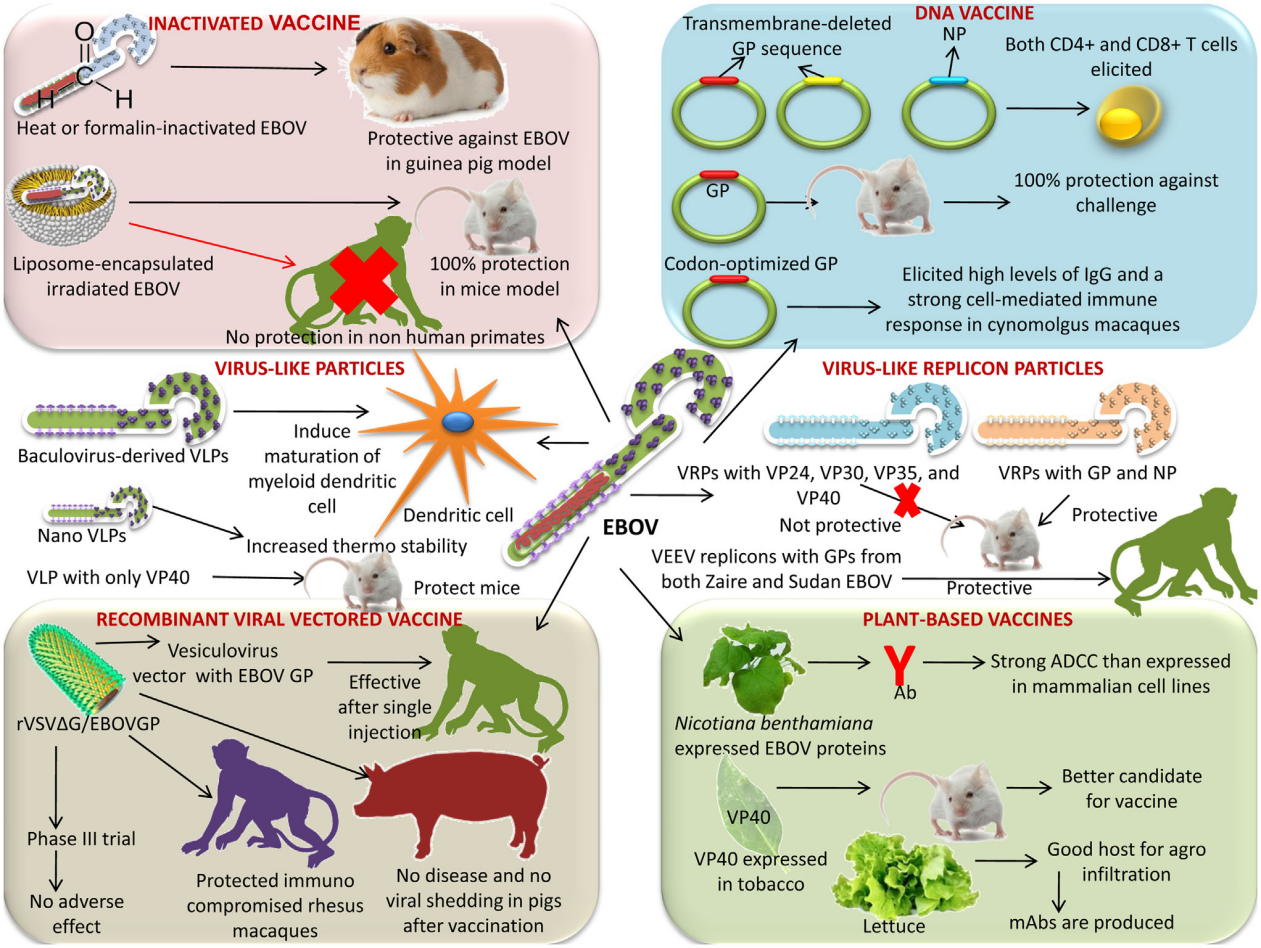
Various vaccine platforms under progress for the development of a successful Ebola virus (EBOV) vaccine. Platforms like inactivated vaccine, DNA vaccine, virus-like particles, virus-like replicon particles (VRPs), plant-based vaccine, and recombinant viral-vectored vaccines are available.

An overview of various types of vaccines for countering EVD is presented in Table 1 and depicted in Figure 1.

**Table 1:** Vaccines for treating Ebola virus disease.

S. No.	Type of vaccine platform	Vaccine	Adjuvant/mode of delivery	Model	Antigen	Inference	Reference
1	Inactivated vaccine	Rabies virus based on inactivated vaccine (FILORAB1)	Glucopyranosyl lipid A	Cyanomolgus and rhesus monkeys	GP	100% protection against lethal Ebola virus (EBOV) challenge, with no to mild clinical signs of disease	Johnson et al. (108)

		Virulent EBOV	Formalin inactivation/heat inactivation	Guinea pig	Complete virus as antigen	Reduction in mortality	Lupton et al. (53)

2	Attenuated vaccine	Live replication-competent EBOV and rabies virus-based bivalent vaccine	Direct inoculation of live-attenuated vaccine	Rhesus macaques	GP	100% protection from lethal challenge	Blaney et al. (109)

3	DNA vaccine	Multiagent filovirus DNA vaccine containing GP of Zaire, Sudan, and Marburg virus (MARV)	Electrical stimulation at an amplitude of 250 V/cm using TriGrid™ electroporation device	BALB/c mice	GP	100% protection from lethal challenge	Grant-Klein et al. (110)

					Mutant GP		

		Synthetic polyvalent-filovirus DNA vaccine against Zaire, Sudan, and MARV	pVAX1 mammalian expression vectors, injected intradermally with 200 µg DNA	Guinea pigs	Codon-optimized GP	100% protection from lethal challenge	Shedlock et al. (111)

		DNA vaccine against EBOV	Intramuscular electroporation (IM-EP) 500 µg dose	Rhesus macaques	Codon-optimized GP	86% protection	Grant-Klein et al. (59)

		DNA encoding Zaire and Sudan glycoproteins	4 mg dose in 1 ml volume	Human healthy adults	Wild-type GP	Antibody response to the Ebola Zaire glycoprotein generated	Kibuuka et al. (60)

4	mRNA vaccine	mRNA molecule encapsulated in a lipid nanoparticle (LNP) formulation	0.2 mg/ml	Guinea pigs	A human Igκ signal peptide or the wild-type signal peptide sequence of GP attached to GP	Potency of mRNA vaccines is enhanced by LNP	Meyer et al. (49)

5	Ebola virus-like particles (VLPs)	pWRG7077 plasmid vectors encoding for Ebola VP40 and GP	10 µg of eVLPs	Balb/c mice	GP and matrix protein (VP40) in mammalian cells	Dose-dependent protection against lethal challenge	Warfield et al. (112)

		MARV GP and EBOV VP40 or vice-versa	Intramuscular vaccination with 100 µg of VLPs + 200 µl RIBI adjuvant	Strain 13 guinea pigs	GP and VP40	Homologous GP is essential and sufficient for protection against lethal challenge with homologous virus	Swenson et al. (113)

		pWRG7077 plasmid vectors encoding for GP, NP, and VP40	3 intramuscular injections of 250 µg of eVLPs + 0.5 ml of RIBI adjuvant	Cynomolgus macaques	GP, NP, and VP40	All animals were protected without showing signs of clinical illness	Warfield et al. (114)

		293T cells transfected with	VLP containing 10 µg GP	C57BL/6 mice	GP + VP40	VLP-mediated anti-EBOV immunity in B cell-deficient mice	Cooper et al. (66)

6	Vaccinia virus-based vaccine	Modified vaccinia virus Ankara-Bavarian Nordic^®^ (MVA-BN) co-expressing VP40 and glycoprotein (GP) of EBOV Mayinga and NP of Taï Forest virus	Intramuscular or intravenous application of 10^8^ TCID_50_ of MVA-BN-EBOV-GP or MVA-BN-EBOV-VLP	CBA/J mice	GP + VP40	Production of non-infectious EBOV-VLPs	Schweneker et al. (95)

		Modified vaccinia Ankara (MVA)-based vaccine expressing the EBOV-Makona GP and VP40	1 × 10^8^ TCID_50_	Rhesus macaques	GP + VP40	100% protection with single or prime/boost vaccination	Domi et al. (115)

7	Venezuelan equine encephalitis virus (VEEV)-based vaccine	VEEV-like replicon particles (VRP)	10^7^ IU VRP	Strain 2 or strain 13 guinea pigs	NP or GP	NP-VRP and GP-VRP immunized animals completely protected against lethal challenge	Pushko et al. (116)

		VRP expressing SUDV GP + EBOV GP	10^10^ focus-forming units	Cynomolgus macaques	GP (EBOV + SUDV)	100% protection against intramuscular challenge with either SUDV or EBOV	Herbert et al. (68)

8	Cytomegalovirus (CMV)-based vaccines	CD8^+^ T cell epitope from EBOV NP (VYQVNNLEEIC) cloned in mouse CMV vector	5 × 10^5^ plaque forming units	C57BL/6 mice	NP	High levels of long-lasting (>8 months) CD8^+^ T cells are produced	Tsuda et al. (117)

9	Kunjin virus-based vaccine	Kunjin virus VLPs expressing GP	5 × 10^6^ VLPs	Dunkin–Hartley guinea pigs	GP	More than 75% survival of animals post challenge	Reynard et al. (118)

10	Paramyxovirus-based vaccines	Human parainfluenza virus type 3 (HPIV3) clone containing GP	10^7^ plaque-forming units	Rhesus monkeys	GP	Double immunization protected animals	Bukreyev et al. (119)

		Newcastle disease virus clone containing GP	10^7^ plaque-forming units	Rhesus monkeys	GP	NDV/GP is highly attenuated for replication in the respiratory tract of immunized animals and developed GP-specific mucosal IgA antibodies	DiNapoli et al. (120)

11	Adenovirus-based vaccines	Adenovirus (rAd5) vaccine GP	2 × 10^9^ virus particle	Phase I human study	GP	Antigen specific humoral and cellular immune responses were generated	Ledgerwood et al. (121)

		Adenovirus (ChAd3) vaccine boosted with MVA	Priming dose 2.5 × 10^10^ PFU of ChAd3 and a boosting dose of 1.5 × 10^8^ PFU of MVA	Healthy adult volunteers	GP	Elicited B-cell and T-cell immune responses	Ledgerwood et al. (91)

		Chimpanzee serotype 7 adenovirus vaccine expressing GP (AdC7-GP)	Prime boosting with AdC7-GP (1 × 10^10^) and boosting with 20 mg *Drosophila* S2 cells expressed truncated GP	BALB/c mice	GP	Long-lasting high-titer neutralizing antibodies production in mice and efficiently prevented luciferase-containing reporter EBOV-like particle entry even at 18 weeks post-immunization	Chen et al. (90)

12	Vesicular stomatitis virus (VSV)-based vaccines	VSV GP replaced with EBOV GP	2 × 10^7^ PFU	Healthy adult volunteers	GP	Anti-Ebola immune responses were documented	Regules et al. (82)

		VSV GP replaced with EBOV GP	3 × 10^5^ PFU	Healthy adult volunteers	GP	Lowered antibody responses observed with vaccine associated side effects like vaccine-induced arthritis and dermatitis	Agnandji et al. (122)

13	Semliki forest virus based vaccines	From DNA-launched replicons (DREP)-eGFP vector, eGFP replaced with GP and NP to make DREP-GP and DREP-VP40 vectors, respectively	10 µg plasmid DNA	Balb/c mice	GP + VP40	EBOV filamentous VLPs were observed in the supernatant of cells resulting from co-expression of GP and VP40 and post immunization, specific humoral accompanied with a mixed Th1/Th2 cellular immune response was obtained	Ren et al. (69)

14	Liposome-encapsulated vaccine	Liposome-encapsulated irradiated EBOV-Zaire (6 × 10^6^ rads of γ-irradiation from a ^60^Co source)	Intravenous inoculation of 1.0 ml dose containing 194 µg of irradiated EBOV Zaire + 100 µg of lipid A	BALB/c mice and Cynomolgus monkeys	All native EBOV antigens	All mice protected, however the immunization failed to protect Cynomolgus monkeys	Rao et al. (54)

## Advances in Developing Drugs and Therapies Against EBOV

Momentous leap has been witnessed toward the designing of efficacious EBOV drugs and therapeutics during the short span of only few years, even though the efficacy of several biologicals and vaccines were evaluated during the recent West African outbreak, it remained elusive to ratify a licensed EBOV disease treatment regimen (123).

Management of suspected or confirmed EVD patients includes quarantine, symptomatic, and supportive treatments, including fluid replacement, electrolyte imbalance correction, treating complicated infections, and preventing shock (124). For mitigation of the huge fluid loss and resultant hypovolemia, oral rehydration solutions should be provided adequately and if required anti-diarrheal and anti-emetic drugs need to be administered (125). Brincidofovir, a drug used to treat dsDNA viruses such as adenovirus, herpesviruses, orthopoxviruses, papillomavirus, and polyomaviruses, was approved for emergency treatment of two patients with EBOV infection; however, the clinical efficacy of the drug is unknown (126). Many drugs are being tested to identify specific antiviral drugs to treat EBOV, and new drug candidates are being developed by researchers worldwide (26, 127, 128).

Favipiravir (T-705), an antiviral drug found useful in treating influenza, has been studied and found effective against EBOV (129–131). Insertional mutagenesis, a high-throughput method to identify genes responsible for virus replication, can be used to develop drug candidates (132). Molecular docking experiments with EBOV GPs can be used for drug designing and the development of therapeutics (133, 134). Novel flexible nucleosides called fleximers were found to be effective against recombinant EBOV in Huh7 cells (135). Ribavirin antiviral can be recommended for the treatment of EBOV, since in mouse and monkey models, treatment with ribavirin delayed the death and increased survival rate (136). However, adverse effects associated with its use may limit ribavirin use (137). Lamivudine, an anti-retroviral drug, has been tested by Liberian doctor on 15 EBOV patients with survival of 13 patients (138). However, study by Cong et al. (139) found no survival benefits in Guinea pig model. Similar results were obtained by Hensley et al. (140), with no significant antiviral activity of lamivudine against EBOV in Vero E6 cells. Hence, the use of lamivudine may not be advocated.

The docking of VP40, VP35, VP30, and VP24 has been achieved using small molecules belonging to the class of flavonoids and derivatives. Gossypetin and Taxifolin (the two flavonoids of top ranks) showed higher docking scores for every EBOV receptor (141). A virtual analysis of more phytochemicals could help to identify plant-derived products with comparatively higher efficacy and lower toxicity. Adenosine nucleoside analogs such as BCX4430 have been found effective against EBOV in a mouse model. GS-5734, also a nucleoside analog, was found effective against EBOV in a NHP model (142). Using molecular dynamics simulations, graphene sheets are found to associate strongly with VP40 (matrix) protein of EBOV and disrupt VP40 hexamer–hexamer association, crucial to form virus matrix, thereby graphene and similar nanopolymers may be used as therapy or at least disinfectant to reduce the risk of transmission at time of epidemic (143).

The potential of retro-type drugs (molecules that block the retrograde trafficking of bacterial and plant toxins within mammalian cells) must be explored for designing novel therapy against filovirus (144). Retro-2 along with its other two derivatives, Retro-2.1 and compound 25 could effectively block EBOV and MARV progression *in vitro*. The derivatives were shown to be more potent inhibitors of filoviral penetration, replication, and progression when compared with their parent compound, as evidenced by pseudo-typed virus assays (144).

### EBOV Entry and Inhibitors

The cell entry of EBOV involves virus binding to the cell surface receptors followed by internalization through macropinocytosis, processing by endosomal proteases, and transport to Niemann–Pick C1 (NPC1; an internal receptor for EBOV) containing endolysosomes. Phosphatidylinositol-3-phosphate 5-kinase is essential for maturation of endosome, a critical step to the EBOV infection (145). *In vitro* studies using apilimod, an antagonist of phosphatidylinositol-3-phosphate 5-kinase, showed inhibition of EBOV by blocking the viral particle trafficking to NPC1 containing endolysosomes (146). IFNs are natural antivirals, and type I (IFN-α/β), particularly, is being widely used for the treatment of viral diseases. Type I IFN-α2b has been evaluated for the treatment of EBOV; however, IFN-α2b was not successful, as only delayed death but could not prevent mortality in EBOV-exposed monkeys. IFN-γ reduced the mortality rate in mice when administered either before or after EBOV infection (147), suggesting its promise as a prophylactic and/or therapeutic drug for use in EBOV infections. As IFN-γ has already been used to treat certain chronic medical conditions and has been approved by the FDA, it can be readily adapted for use against EBOV infections.

Silvestrol extracted from *Aglaia foveolata* was found to inhibit replication of EBOV and has been suggested as a therapeutic drug to treat acute EBOV infection (148). Supportive treatments like oral rehydration therapy are recommended for children under 5 years of age (149). Web-based identification of therapeutic agents indicated that a single siRNA can inhibit mRNA transcription of three species of EBOV, whereas 75 siRNAs can inhibit at least two species of EBOV. The web server Ebola VCR has been developed, with details available for the development of suitable therapeutic agents (150). Numerous treatment options for EVD are discussed below.

Ebola virus possesses only one surface protein and is responsible for both the receptor binding and fusion of virus-to-host cell endosomal membrane. EBOV glycoprotein binds with lectin receptor DC-SIGN (151). The infection initiated with the binding of EBOV glycoprotein to lectin receptors and internalization of virus majorly through macropinocytosis and as alternative mechanism through clathrin-dependent endocytosis (152). In the low pH of endosome, cysteine proteases including cathepsin B and L proteolytically cleave GP. Possibly this proteolytic cleavage exposes the putative receptor-binding region that interacts with NPC1, a receptor facilitating the filovirus entry (153). TIM-1 receptors directly interact with phosphatidylserine on the viral envelope, suggestive of GP independent virus attachment onto the cells. In poorly permissive cells, EBOV infection enhanced by exogenously expressed TIM-1 by 10- to 30-folds (154). Other phosphatidylserine interacting proteins like TIM-4 and Axl (a receptor tyrosine kinase) also have been demonstrated to enhance the infection of several enveloped virus. A benzylpiperazine adamantane diamide-derived compound obtained after screening of a library of small molecules, targets endosomal NPC1, and inhibit infection by VSV particles (VSV) pseudo-typed with EBOV GP (155).

Tetrandrin is a potent drug that inhibits the EBOV entry into the cells (156). Two estrogen receptor drugs, clomiphene and toremifene, have been reported to hinder EBOV infection in mice by blocking cell entry and fusion with host cells (157). Amiodarone, an ion channel blocker, has been found to inhibit EBOV entry into cells (158, 159). Dendrimers and fullerene C_60_ have unique symmetrical properties and were recently found effective in inhibiting EBOV entry *in vitro* (160). Clarithromycin, an antibiotic, inhibits the release of calcium (stimulated by nicotinic acid adenine dinucleotide phosphate) from lysosome and exhibit anti-EBOV activity. Alike clarithromycin, posaconazole, an anti-fungal agent also shows similar anti-EBOV activity. In addition, it also inhibits the functions of NPC1 protein and acid sphingomyelinase activity. Both drugs, i.e., clarithromycin and posaconazole, ultimately inhibit the entry of EBOV into the host cell (161). The drug 5-(*N*-ethyl-*N*-isopropyl) amiloride inhibits the process of macropinocytosis (a process required for uptake of large filamentous virions, like EBOV) and thus interferes with the viral entry into the cell. Compounds like MLS000394177 and MLS000733230 also inhibit the viral entry into cells (162).

*Prunella vulgaris*, a Chinese herb, was found to inhibit EBOV entry into cells, using an EBOV-GP-pseudotyped-human immunodeficiency virus (HIV)-1-based vector system (163). Pseudovirions containing EBOV-GP were used for screening of the Prestwick chemical library, which contains 1,200 FDA approved drugs. The assay was based on cell entry of HIV-1-based surrogate in 384-well format. Twenty chemicals were found to inhibit more than 80% entry and 16 out of them were identified as G protein-coupled receptor (GPCR) antagonists, which target a range of GPCRs including adrenergic receptors, 5-HT (serotonin) receptors, histamine receptors, muscarinic and acetylcholine receptors. The time-of-addition studies suggested that EBOV entry is stopped at level of initial attachment prior to fusion of virus and cell membrane (164). Quercetin 3-β-*O*-d-glucoside (Q3G), a flavonoid derivate, was found to protect mice against EBOV challenge by targeting viral entry (165).

During replication of EBOV, surface GPs undergo proteolytic cleavage in the endosome by several proteases, including cathepsin B (CatB) (166). Thus, proteases may be a good target for the inhibition of EBOV replication. One study showed that, using a synthetic serine protease inhibitor, nafamostat mesilate (NM), caused a reduction in CatB release in rat pancreases. NM was also found to have anti-coagulant properties, which would also be useful in EBOV infections, as EBOV causes disseminated intravascular coagulation. Thus, this drug should be examined in clinical trials to be approved for the treatment of EVD (167). Chemically modified human serum albumin with 3-hydroxyphthalic anhydride (HP-HSA) has been demonstrated with the potential of a therapeutic candidate in resisting the EBOV infection (168).

### Transfusion of Convalescent Blood/Serum

Convalescent serum by definition contains immunoglobulins IgM and IgG but is devoid of red blood cells and clotting factors. Transfusing convalescent whole blood and convalescent plasma from disease survivors has been found to neutralize EBOV and reduce its load; thereafter, the immune response of the patient can provide protection against EBOV (169, 170). The use of whole blood and convalescent serum was approved by the World Health Organization (WHO) during critical EBOV conditions (171, 172). Screening of plasma is needed to rule out the presence of residual EBOV RNA and other blood-transmitted pathogens such as HIV, hepatitis B virus, and hepatitis C virus. Protection is conferred in NHPs through antibody therapy (post-exposure). In humans, this has ultimately paved the way for filovirus therapy by the use of polyclonal/mAb (approved by Food and Drug Administration) (173).

Valuable emergency therapeutics for the treatment of EBOV-infected persons include passive immunization with neutralizing antibodies by the transfer of sera from individuals recovering from EVD (174, 175), although it is not considered to render 100% protection especially after exposure (3 days post-exposure) to EBOV (e.g., Zaire Ebolavirus Makona) (176). Precise immunoglobulins retrieved from equine serum against EBOV were found safe and effective as prophylactic therapy in non-allergic patients (177). Recently developed mAb-based treatments for EVD include mAb114 and MB-003, ZMAb, ZMapp, and MIL-77E cocktails (25). ZMAb, consisting of three murine mAbs (1H3, 2G4, and 4G7), administered at a dose of 25 mg/kg three times, completely protected cynomolgus macaques against EVD. Administration of ZMAb with adenovirus-vectored IFN-α resulted in 75 and 100% survival of cynomolgus and rhesus macaques, respectively (29). mAbs that bind to the base of GP (4G7 and 2G4) are neutralizing antibodies, whereas mAbs that bind to the glycan cap (mAb114, 1H3, and 13C6) are non-neutralizing antibodies. The chimeric human mAbs 13C6, 6D8, and 13F6 possess the variable region from mice and Fc region of human; mAbs 13C6 and 6D8 neutralize EBOV in the presence of complement proteins (24). By repeated immunization of mice with glycoproteins of filovirus, generation of pan-EBOV-specific (as well as pan-filovirus) mAbs have been obtained. These pan-EBOV mAbs have shown reaction with RESTV, SUDV, and other viruses (178).

The components of ZMapp are mAbs (chimeric), *viz*., c13C6 from MB-003 (already known cocktail of antibody) and c2G4 as well as c4G7 from ZMab (different cocktail of antibody). This drug reversed clinical signs in 100% of rhesus macaques, even when administered as late as 5 days after EBOV exposure (30). Use of ZMapp, humanized-mouse antibodies, as a therapeutic agent has shown promise in NHPs (30, 84, 179) and it is a WHO-approved treatment regimen for EVD. Recently, a baby born to an EBOV-infected mother was found positive for EBOV on the first day of life. The baby was treated with ZMapp and the broad spectrum antiviral GS-5734, and, on day 20, the baby was found negative for EBOV (180). MB-003 is another mAb cocktail which is found to be effective in NHPs against variants of EBOV that are resistant to ZMapp (181).

The mechanism of action of mAbs is that they identify the inter-protomer epitope of the GP fusion loop, which is essential for viral membrane fusion, and also neutralize the entry of virus (182). Although several mAbs are available that can neutralize EBOV, there are few mAbs that can neutralize GPs from different EBOV species. In a study by Duehr et al. (183), a panel of eight murine mAbs derived from animals immunized with *Zaire ebolavirus* was evaluated. The mAbs were tested for binding breadth using a set of recombinant surface GPs from RESTV, TAFV, BDBV, EBOV, SUDV, and MARV. Of the eight, two mAbs (KL-2E5 and KL-2H7) showed binding ability. These two mAbs did not neutralize EBOV; however, they protected mice from infection with a VSV expressing the *Zaire ebolavirus* GP. Duehr et al. (183) also suggested that Fc-FcR interactions are responsible for the protection of mice in the absence of neutralization. Although ZMapp was found to be effective against Zaire EBOV, it has not shown cross-protection against other species of EBOV. FVM04 (a mAb) has shown cross neutralizing activity against SUDV. So, it can be used to replace one of the components of ZMapp, thereby increasing the range of protection against SUDV, ultimately leading to generation of cross-protective mAbs cocktail (184).

One Fab, KZ52, obtained by panning of phage display library, was derived from the bone marrow of an EVD survivor. Fab KZ52 exhibited 50% neutralization at a concentration of 8 nM (185). The mAb KZ52 protected guinea pigs from lethal *Zaire ebolavirus* challenge; however, when an experiment was carried out in rhesus macaques, the antibody failed to protect animals prophylactically and did not inhibit viremia (186). EBOV GP is processed by cathepsins, and the cleaved GP fuses with host cells to form a fusion pore, a passage for the EBOV genome to enter the cytosol for replication. Human mAb KZ52 and monkey mAb JP3K11 bind to conformation-dependent epitopes of GP. KZ52 is directed to bind a conformational non-glycosylated epitope at base of GP and a total 23 residues of GP residues remain in contact with antibody. Out of 23, 15 are contacted through van der Waals interactions and remaining 8 through direct hydrogen bonds (187). At 0.4 µg/ml dose, KZ52 lead to 50% neutralization. KZ52 protective efficacy is due to inhibition of cathepsin mediated cleavage of GP (23).

Exploring the synergistic effect of different pairs of neutralizing and non-neutralizing anti-EBOV mAbs could provide 100% protection in mice, revealing the scope of this approach in designing and developing immunotherapeutics and vaccines (188).

Bispecific Trojan-horse antibodies neutralizing other filoviruses have been found to provide protection in mice from multiple EBOV infection (189). Cell-penetrable human scFvs (HuscFvs) (transbodies) that bind to EBOV VP40, a matrix protein pivotal for viral assembly and budding, produced by phage display technology, revealed inhibition of the EBOV-like particles (VLPs) egress from hepatic cells (190). These transbodies were effective in blocking viral assembly and budding within the cells as they bind to several cationic patches in the VP40 C-terminal domain. The transbodies inhibit the function of VP40 by additional mechanisms also; such as binding to N-terminal domain and L-domain peptide WW binding motifs, suggesting the potential of these transbodies as direct acting anti-EBOV agents in future (190). Cell-penetrable HuscFvs specific to a highly conserved interferon-inhibitory domain (IID) of VP35 of EBOV inhibited the VP35 biofunctions in the EBOV replication cycle including polymerase cofactor activity and host IFN–antagonism by forming interface contact with residues of the first basic patch, the central basic patch, end-cap, and residues important for IID multimeric formation for dsRNA binding (191). The cell-penetrable small antibody fragments (HuscFvs) or superantibodies [the term coined by Kohler and Paul (192)] can cross the membrane of all cells but get accumulated intracellularly only where the target antigen is present. Thus, disappearance of the superantibodies from the blood circulation does not imply that they are eliminated from the body. The transbodies to the highly conserved EBOV VP40 and VP35 should be evaluated further using authentic EBOV in animal models of EVD and clinical trials before they can be considered a broadly effective and promising alternative to existing treatment approaches for EVD.

Recently, three mAbs produced in tobacco plants that target the EBOV GP were tested and showed good results in humans (193). Human mAbs against BDBV GP were isolated from patients who survived during the 2007 Uganda outbreak. These mAbs were found to have a neutralizing effect against multiple EBOV species, suggesting the possibility of the use of single mAbs as cross-protecting antibodies (194). Another investigation showed that EBOV GPs were conserved across different EBOV species. ELISA revealed that four mAbs namely S3, S12, S17, and S33 were found to show cross-reaction with GPs of five different species of EBOV (195). The discovery of cross-protective antibodies can aid in the development of therapeutic strategies for treatment of EBOV disease (196). In another study, 349 EBOV GP mAbs were isolated from survivors of EVD in an outbreak in Zaire, and 77% of the mAbs were found to neutralize EBOV (197). Three mAbs of EBOV-GP (Q206, Q314, and Q411) were isolated during the West African EVD outbreak in 2014. Recognition of the novel epitopes has been performed for Q206 and Q411, wherein these mAbs were found protecting mice against EBOV (198). A therapeutic vaccine based on mAbs has been proposed to sufficiently resolve replication of invasive EBOV, even if administered as a single dose 4 days post-infection (199). Non-neutralizing mAb 5D2 or 7C9 expressing adeno-associated virus (AAV), consistently released mAb in body and was found 100% protective against mice adapted EBOV strain. Neutralizing mAb 2G4 conferred 83% protection and a cocktail of these two mAbs provided 100% protection when given 7 days prior to infection and sustained protection when immunized animals were challenged 5 months post AAV-mAb immunization (200).

Potential limitations of mAb-based therapies include the requirement for high doses and mAb mixtures that are outbreak-specific owing to constant viral evolution. Furthermore, epitope mutations could reduce efficacy of the therapeutic mAbs used. Hence, these limiting factors need to be taken care of accordingly with mAbs usages.

### EBOV Gene Expression Inhibitors

Viral gene expression is dependent on host cell machinery and is critical for virus replication. A conserved guanine-rich sequence in the EBOV L gene has been reported to assemble into quadruplex RNA, targeted by cationic porphyrin TmPyP4 that directs inhibition of the expression of L gene at the RNA level (86, 201). BCX4430 (a nucleoside analog) is a viral RNA polymerase inhibitor, and it has been found effective in protecting mice against lethal challenge of EBOV (202–204). In addition, double-stranded RNA binding protein 76 has been reported to inhibit EBOV polymerase activity (205).

Small molecular inhibitors needed for the synthesis of polyamine have been found to block the expression of EBOV gene. The eukaryotic initiation factor 5A (eIF5A) hypusination and spermidine (a polyamine) are essentially required for the replication of EBOV. However, if eIF5A hypusination is blocked, the gene expression of EBOV is inhibited which subsequently blocks the replication of the virus. Therefore, in-depth understanding of this mechanism at molecular level is essential for developing anti-EBOV drugs (205).

### Repurposed Drugs

It is time taking task to develop a new therapeutic against an infectious agent and till that time new therapy divulged; drug repurposing, *i.e*., already existing drugs may be screened for their efficacy against pathogen. Owing to the lack of approved EBOV therapies, the screening of potentially efficacious drugs revealed that few of the drugs could be repurposed for EBOV treatment (206) (Table 2). Amiodarone, dronedarone, and verapamil, which are used for tachycardia, arrhythmias, and high blood pressure or angina, respectively, have been screened for their ability to inhibit the entry of filoviruses into cells and found efficacious in *in vitro* models (158). The use of statins, angiotensin-converting enzyme inhibitors, and angiotensin receptor blockers has also been suggested to attenuate EBOV infection (126). Phosphoinositide 3-kinases inhibitor LY294002 and calcium/calmodulin kinase (CAMK2) inhibitor KN-93 have been reported to reduce EBOV infection in Vero E6 cells. The p38 mitogen-activated protein kinase inhibitor SB202190 was shown to check virus-mediated cytokine storm, as studied in monocyte-derived DC of humans (207). Estrogen re-uptake modulators, *viz*., toremiphene and clomiphene, although cause *in vitro* inhibition of the virus entry, but are not free from unwanted side effects like ocular adverse reaction (in case of clomiphene) and serious derangements of electrolytes (in case of toremiphene) at the higher doses. To overcome this, combination therapy is suggested while using such drugs (206). Brincidofovir, a cidofovir analog conjugated with a lipid, can prevent EBOV replication; however, its exact efficacy in an *in vivo* model needs to be determined (208). Because cyclophilin A (CypA) is not essential for EBOV replication, alisporivir, which inhibits the host protein CypA, has shown limited antiviral effects against EBOV strains (Makona, Mayinga) (209). Emetine, an anti-protozoal agent, and its desmethyl analog cephaeline have potently inhibited EBOV replication and cephaeline is well tolerated in patients than emetine (210).

**Table 2:** Repurposed drugs used in Ebola virus disease therapy.

S. No.	Method of screening	Repurposed drug	Function of drug	Reference
1	*In vitro* antiviral activities	Verapamil	Hypertension, angina and arrhythmia	Gehring et al. (158)

		Teicoplanin (block a late stage of viral entry)	Glycopeptide antibiotic	Wang et al. (211)

		Nocodazole	Cell arrest in G2- or M-phase	Yonezawa et al. (212)
		Cytochalasin B	A mycotoxin, inhibits network formation by actin filaments	
		Cytochalasin D	Induces depolymerization of actin filaments	
		Latrunculin A	Microtubule inhibitor	
		Jasplakinolide	Stabilization of filamentous actin	

		FGI-104	Anti-malarial	Kinch et al. (213)

		Amodiaquine	Anti-malarial	Ekins et al. (214)

		Chloroquine (CQ)	Anti-malarial	Madrid et al. (215); Salata et al. (216)
		Amiodarone	Anti-arrhythmic	
		Prochlorperazine	Dopamine (D2) receptor antagonist; an antipsychotic agent	
		Benztropine	For treating Parkinson’s disease symptoms including muscle spasms, stiffness, tremors, sweating, drooling, and poor muscle control	
		Azithromycin	Macrolide antibiotics	
		Chlortetracycline	Antibiotics	
		Clomiphene	Induce ovaries to produce two or three eggs per cycle	

2	A high-throughput assay for Zaire Ebola virus (EBOV) has been developed using the recombinant EBOV engineered to express the enhanced green fluorescent protein (eGFP) (interfere with viral fusion-worked in *in vitro* and *in vivo*)	Clomiphene		Johansen et al. (157)
		Toremifene	For treating gynecomastia	

3	Mice model (50–90% survival)	Yuan (217)

4	*In vivo* murine EBOV infection model	Bepridil	Calcium channel blocker	Johansen et al. (218)

		Sertraline (block a late stage of viral entry)	Antidepressant	Johansen et al. (218)

5	Recombinant vesicular stomatitis virus containing Ebola GP protein	Tunicamycin	A nucleoside antibiotic	Takada et al. (219)

6	In human—single-arm proof-of-concept trial in Guinea pigs	Favipiravir	Broad-spectrum antiviral activity against RNA viruses	Sissoko et al. (220)
	Mouse model (100% protection)			Oestereich et al. (221)

7	Vero E6 cells infected with infectious Mayinga strain of EBOV	Amiodarone	Anti-arrhythmic therapy and multiple ion channel blocker	Gehring et al. (158)
		Dronedarone	Anti-arrhythmic therapy	
		Verapamil	Ca^+2^ channel blocker	

8	Primary human monocyte culture	17-allylamino-17-demethoxygeldanamycin (17-AAG)	Inhibitor of heat-shock protein 90	Smith et al. (222)

9	Recombinant EBOV variant Mayinga expressing enhanced GFP	Retro-2, Retro-2.1, and compound 25	Inhibit EBOV cell entry	Shtanko et al. (144)

10	In 293T cells release of Ebola virus-like particles (VLPs) assay	Nilotinib	Treatment for chronic myeloid leukemia in chronic phase	García et al. (223)

11	Mouse infection model and Ebola VLP entry assay	Clomiphene	Induce ovaries to produce two or three eggs per cycle	Nelson et al. (224)

12	Ebola VLP entry assay	Vinblastine	Microtubule inhibitors	Kouznetsova et al. (225)
		Vinorelbine		
		Vincristine		
		Colchicine		
		Nocodazole		
		Mebendazole		
		Albendazole		
		Tamoxifen	Estrogen receptor modulators	
		Raloxifene		
		Clemastine	Antihistamine and anticholinergic activities	
		Maprotiline		
		Benztropine		
		Clomipramine	Antipsychotic/antidepressant	
		Thiothixene		
		Trifluoperazine		
		Dronedarone	Pump/channel blocker	
		Digoxin	Anti-arrhythmic drug	
		Dronedarone		
		Propafenone		
		Sunitinib	Receptor tyrosine kinase (RTK) inhibitor	
		Daunomycin	Cancer treatment	
		Clarithromycin	Macrolide antibiotic	

13	Ebola live virus assays	Posaconazole	Invasive aspergillosis and candidiasis treatment	Sun et al. (161)

14	Laboratory animal model C57BL/6 and BALB/c mice	Chloroquine and amodiaquine	Anti-malarial and anti-inflammatory	Madrid et al. (226)

15	Small molecule chemical screening	NSC 62914	Scavenger of reactive oxygen species	Panchal et al. (227)

16	Rhesus macaque model of Ebola hemorrhagic fever	Recombinant nematode anti-coagulant protein c2	Inhibitor of blood coagulation, attenuates the proinflammatory response	Geisbert et al. (228)

17	Vero E6 cells infected with Mayinga strain of Zaire EBOV	Suramin	Trypanosome-caused river blindness treatment	Henß et al. (229)

18	Computational analysis using Surflex, PLANTS, AutoDock, and AutoDock Vina	Indinavir	Human immunodeficiency virus protease inhibitor	Zhao et al. (230)
		Sinefungin	Anti-fungal	
		Maraviroc	Antiviral drug	
		Abacavir		
		Telbivudine		
		Cidofovir		

19	Computational analysis of novel drug using CANDOCK (have shown anti-EBOV potential in other modalities also)	Raloxifene	As described above in the table	Chopra et al. (231)
		Tamoxifen		
		Clemastine		
		Deslanoside		
		Digoxin		
		Mebendazole		
		Sertraline		
		Niclosamide		
		Sertraline		

Rosuvastatin, atorvastatin, and pravastatin have been reported to alleviate inflammation, reduce C-reactive protein and TNFα levels, and impede cholesterol-supported EBOV membrane biosynthesis (232). In EBOV infection, overexpression of the procoagulant tissue factor in monocytes and macrophages and participation of endothelial cells leads to an imbalance in coagulation. The use of recombinant nematode anticoagulant protein c2, an inhibitor of tissue factor-mediated blood coagulation, was found to improve survival of macaques from Ebola hemorrhagic fever, and hence suggested to act as a good treatment module targeting the disease development (228). Anti-malarial drugs such as chloroquine and its structural analogs (hydroxychloroquine, pamaquine, primaquine, and plasmaquine) also act as lysosomotropic agents, preventing endosomal/lysosomal acidification, and thus limiting certain viral infections (233). There are conflicting reports on the therapeutic effects of chloroquine in mouse, hamster, and guinea pig models of EVD. Chloroquine was found to inhibit virus replication in *in vitro* studies but failed to protect against EBOV infection and disease development in mice, hamsters, and guinea pigs (234, 235). Esomeprazole and omeprazole were also found to inhibit viral entry during *in vitro* studies but higher concentrations of these drugs may be required when to be used *in vivo* (236). During the EBOV outbreak in Liberia in 2014, a reduction in fever cases was observed following mass administration of malaria chemoprevention drugs (237).

Because of its competitive anti-heparin potential and interference with viral replication and entry into the cell, the anti-trypanosomal agent, Suramin (Germanin or Bayer-205) has been proposed to treat EVD (229). A pyrazine carboxamide derivative namely Favipiravir (an anti-flu medicine), which was used earlier as an inhibitor of influenza virus replication, has been found useful in both therapy and prophylaxis during EBOV epidemic in West Africa (238–240). Favipiravir and the pyrazine carboxamide derivative T-705 showed positive results in treating patients with medium to high viremias, although these drugs were not found to be effective with very high viremias, but revealed acceptable results during EBOV infection in mouse (219, 221, 241).

The microtubule inhibitor drugs (vinblastine, vinorelbine/navelbine, and vincristine), commonly used as anticancer agents, have been found effective in inhibiting EBOV VLP entry into HeLa cells even at low concentrations (48–140 nM). Colchicine, a microtubule modulator primarily used for gout, has also been found to show anti-EBOV activity (225, 242).

Screening of 1766 FDA approved drugs and 259 experimental drugs revealed that Indinavir, an HIV protease inhibitor, may be effective in reducing the severity of EVD (230). The antiviral drugs including Maraviroc, Abacavir, Telbivudine, and Cidofovir could target the MTase domain of EBOV and inhibit the viral RNA-directed RNA polymerase (230). The Computational Analysis of Novel Drug Opportunities platform was recently developed to screen drugs approved by the FDA. Drugs like enfuvirtide, vancomycin, bleomycin, octreotide, lanreotide, somatostatin, and ubidecarenone (CoQ10) have shown higher activity against EBOV (231). Recently, virtual screening of several thousands of repurposing drugs from Drug Bank has been performed and ibuprofen was selected by realizing its possible inhibitory effect on EBOV infection. The drug has been found to show detectable antiviral effect in cell culture and can thus be used as a very useful molecular template for anti-Ebola viral drug development (243).

### Nucleotide Analog Prodrug

GS-5734 developed by Gilead Sciences falls under this category. Interestingly, clinical trials have been conducted and it has been found that the drug is effective in clearing virus from semen (181, 244). Administration of GS-5734 in rhesus monkey through intravenous route resulted in suppression of replication of EBOV. It is also important to note that in NHPs, this compound can provide protection post-exposure (245).

### Interferons

Interferons act as potent inhibitors of EBOV as has been proved through *in vitro* studies conducted involving various types of cells. IFN-β-1a treatment protected mice against a lethal challenge of EBOV (206). The virus clearance from the blood stream is enhanced by IFN β-1a leading to resolution of the symptoms of the disease at an early stage (246). Even though there is increased therapeutic usage of IFNs, but certain side effects are also associated with such treatment, *viz*., fever and myalgia, which must be kept in mind while opting for their use. Moreover, the occurrence of malaria additionally should be ruled out before initiating IFN therapy (206). Tilorone hydrochloride induces IFN response in mice and has been found effective against EBOV due to its action mainly mediated through pathway of innate immunity (IFN related) (247).

### Oligomer-Mediated Inhibition

RNAi and advanced antisense therapies have been reported to provide post-exposure protection against lethal filovirus infections (248). Small interfering (si)RNA targeting RNA polymerase L protein has shown inhibition of EBOV replication and promising results for its use as a post-exposure therapeutic option (249). siRNAs and phosphorodiamidate morpholino oligomers (PMOs) targeting the EBOV RNA polymerase (L) protein protected NHPs against EVD (248, 249). PMOs target co-polymerase protein VP35 and membrane-associated protein VP24 for this protection (250). Antisense PMO-based drugs like AVI 6002, AVI-6003, and LNPs/siRNA (TKM-Ebola) are in clinical trials. TKM-Ebola is a mixture of three siRNAs that target the L, VP24, and VP35 proteins of Zaire EBOV. In NHPs, it efficiently provided post-exposure protection (249, 251). Although the results were promising, clinicians did not use much TKM-Ebola as it could lead to lethal overproduction of cytokines (a dangerous EBOV-induced inflammatory response) (232). A siRNA LNP product named TKM-130803 has been developed for EVD therapy. Although, the infusion of TKM-130803 at a dosage of 0.3 mg/kg/day through intravenous route to adult patients with severe clinical signs of EVD was comparable, it did not show an improved protection to the existing and classical controls (252). LNP-encapsulated short siRNAs protected 100% of rhesus monkeys exhibiting viremia and clinical illness (253). LNP encapsulation is also an effective drug delivery system (127, 253).

The inhibitors of hsa-miR-1246, hsa-miR-320a, and hsa-miR-196b-5p have been found to decrease EBOV GP cytotoxicity during *in vitro* studies; hence, miRNA technology can be used to develop useful therapeutics (254). Antiviral drug AVI-7537, targeting the VP24 gene of EBOV, has been shown to be efficacious in mice and monkeys (255). The EBOV-GP (cleaved) molecule acts as a ligand for NPC1, a transmembrane transfer protein. For entry of the virus into the target cell, an interaction between EBOV-GP and NPC1 domain C is necessary. Two small molecules, a sulfonamide (MBX2254) and a triazole thioether (MBX2270), have been identified as novel EBOV inhibitors suppressing EBOV infection in an *in vitro* model by blocking the entry of virus into target cell *via* inhibiting GP–NPC1 protein interaction (256). This strategy of targeting viral entry could pave way for the development of an anti-EBOV therapeutic agent.

**Figure 2:**
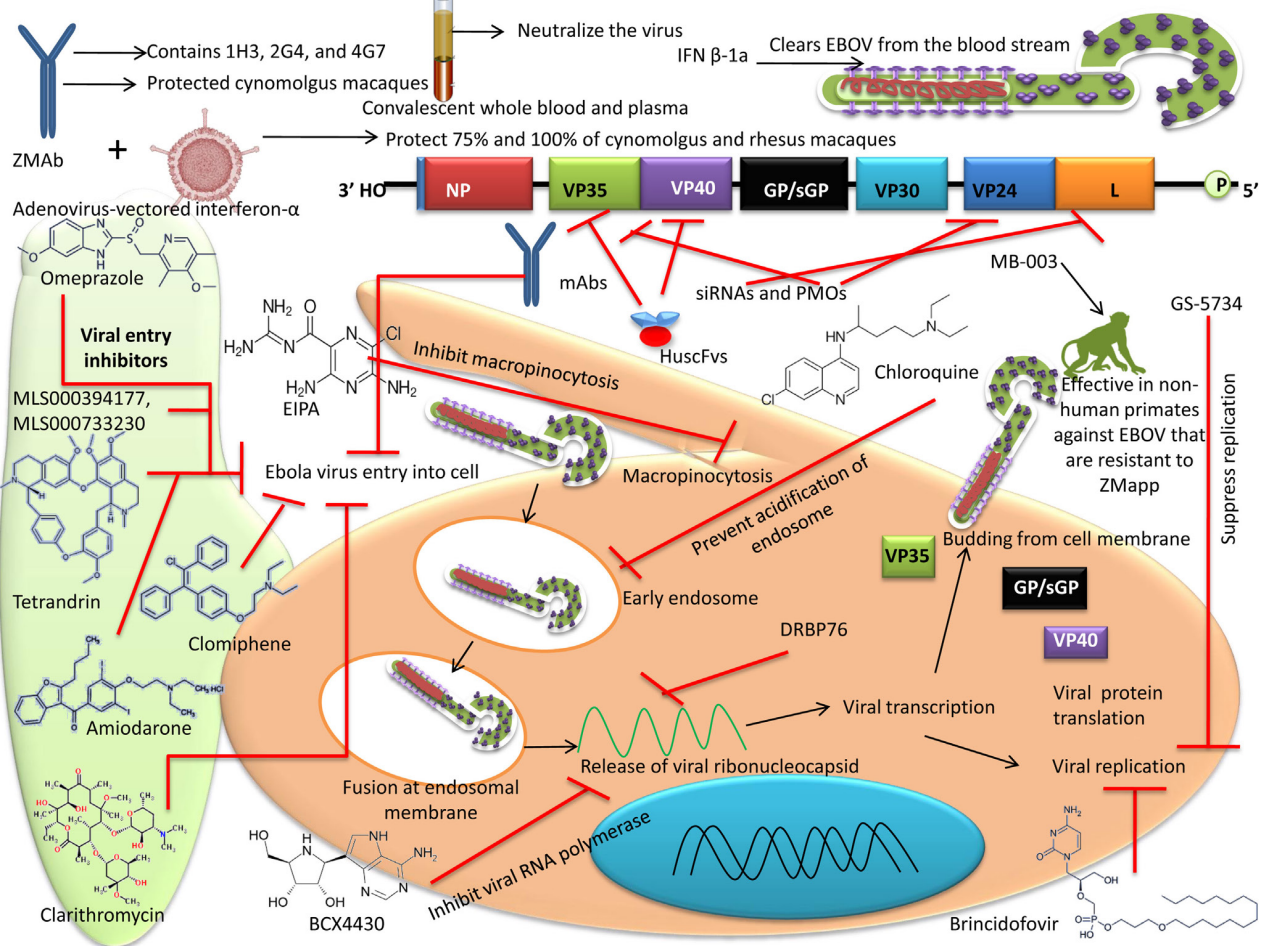
Different therapeutic agents and drugs available for the treatment of Ebola virus disease (EVD). Some agents block the viral entry, some block the RNA polymerase, while some inhibit gene expression. Neutralizing antibodies and mAbs have shown the potential to effectively inhibit Ebola virus (EBOV).

A brief summary of investigated drugs/biomolecules and therapeutics to treat EBOV infection is presented in Table 3 and depicted in Figure 2.

**Table 3:** Investigated drugs/biomolecules to treat Ebola virus (EBOV) infection.

S. No.	Name of the therapy	Name of treatment/biomolecule	Concentration used in experiment	Platform	Experimental model	Inference/notes	Reference
1.	Convalescent blood products therapy	Convalescent whole blood	Transfusion of 150–400 ml blood	–	Human patients	12.5% mortality in treated patients in comparison to 80% in untreated patients	Mupapa et al. (257)

		Polyclonal IgG	Intraperitoneal administration of the purified anti-EBOV IgG (100 mg/kg)	Polyclonal IgG production through trans-chromosomic (Tc) bovine platform technology	Mice	24 h post challenge treatment with SAB-139-V2 antibodies, significant protection was obtained	Dye et al. (258)

2.	Viral entry inhibitors	MBX2254 and MBX2270	MBX2254 (10 µmol/l) and MBX2270 (30 µmol/l) at −1, 0, 2, or 12 h	–	A549 cells	Late stage of EBOV entry is inhibited	Basu et al. (256)

		Tetrandrine	IC_50_ = 55 nM	–	HeLa cells	Inhibits infection of human macrophages, the primary target of EBOV	Sakurai et al. (156)

		MLS000078751 and MLS000534476	8 doses ranging from 0.39 up to 50 µM	Quantitative high-throughput screening (qHTS) approach to screen inhibitor molecules	HeLa cells	Inhibits infection of human macrophages	Anantpadma et al. (162)
		MLS000394177, MLS000730532, MLS000733230				Inhibits early uptake of virus	
		MLS000555232				Inhibits early endocytic trafficking	
		MLS000554255, MLS001101371				Inhibits late endosome trafficking	

		3-hydroxyphthalic anhydride (HP)-modified human serum albumin	EC_50_s for 0.068 and 0.124, respectively, for Zaire and Sudan pseudoviruses	Lentivirus-based pseudotypes	Huh-7 cell	Blocked pseudovirus entry by inhibiting cell surface attachment	Li et al. (168)

		Benztropine mesylate	IC_50_ ranging from 1.7 to 4.9 µM for different strains of EBOV	Pseudo-virions platform for high-throughput sequencing	A549 and vero cells	Screening of Prestwick Chemical Library containing 1,200 FDA approved drugs	Cheng et al. (164)

		*Prunella vulgaris* extract	2.5 µg/ml concentration	EBOV-GP pseudo-typed virus (EBOV-GP-V)-mediated infection model	HEK293T cells	Enhance anti-EBOV activity of the monoclonal antibody mAb 2G4 against EBOV-GP	Zhang et al. (163)

		Quercetin 3-β-*O*-d-glucoside (Q3G)	50 mg/kg of body weight	VSV-EBOV inhibition	BALB/c or C57BL/6 mice (Charles River)	Inhibits glycoprotein-mediated virus entry	Qiu et al. (165)

3.	EBOV gene expression inhibitors	Double-stranded RNA binding protein 76 (DRBP76)	shRNA targeting the 3′ UTR of DRBP76 used^a^	Zaire ebolavirus expressing GFP	293T cells	Inhibits EBOV polymerase activity	Shabman et al. (260)

		Silvestrol	IC_50_ = 96 nM	EBOV-infected human primary macrophages	Huh-7 cells and primary human macrophages	Strong reduction of VP40 levels	Biedenkopf et al. (148)

4.	Interferon (IFN)	IFN-α	IC_50_ = 0.038 µM	*In vitro* model of Ebola Zaire replication with transcription-competent virus-like particles (trVLPs)	HEK 293T cells	Inhibits viral replication 24 h post-infection	McCarthy et al. (261)
		IFN-β	IC_50_ = 0.016 µM				

		IFN β-1a	30 µg/day	Clinical trial	Human patients	Untreated patients had ~1.5- to 1.9-fold more likeliness to die than those treated	Konde et al. (246)

5.	mAb	ZMAb (combination of 1H3, 2G4, and 4G7)	0.1–100 µg/ml	Pseudo-typed VSV platform	VeroE6 cells	Antibodies target the GP1-GP2 interface and the glycan cap	Audet et al. (262)

		ZMapp (cocktail of humanized-mouse antibodies c2G4 and c4G7 and c13C6)	Totaling dose of 5 mg/animal at 1-day post-infection	–	2 patients evacuated from Liberia to Atlanta	Showed promise in non-human primates and clinical improvements in human subjects	Qiu et al. (30)

		KL-2E5 and KL-2H7	10 mg/kg	Pseudo-typed VSV platform	Stat2^−/−^ mice	Non-neutralizing but protective action of mAb due to Fc-FcR interactions	Duehr et al. (183)

		FVM04	Single intraperitoneal (IP) injection of 10 mg/kg	Mouse-adapted EBOV	Mice	At 1 dpi post infection single dosing led to full protection from lethal challenge	Howell et al. (184)

		KZ52	50 mg/kg	Guinea pig-adapted Ebola Zaire virus	Guinea pigs	Dose-dependent protection of guinea pigs and proven record of efficacy in post-exposure prophylaxis of EBOV infection	Parren et al. (186)

		Q206, Q314, and Q411	100 µg of each mAb	Mouse-adapted EBOV	BALB/c mice	Administration of mAbs cocktail at 1 or 2 days post infection, potently neutralized live EBOV	Zhang et al. (163)

		Cell-penetrable human VP40 binding scFvs (HuscFvs)	40 μg/well	Pseudo-typed lentivirus particles carrying EBOV VP40 and GP genes	Huh7 cells transduced with	Human transbodies effectively inhibit egress of Ebola virus-like particles from mammalian cells	Teimoori et al. (190)

		Cell-penetrable human scFvs to IFN-inhibitory domain of VP35	25 μg/well	EBOV minigenome and VP35 expression cassette	HepG2 cells transduced with EBOV minigenome and VP35 expression cassette	Human transbodies effectively inhibit VP35 co-polymerase activity and antagonize VP35-mediated IFN suppression	Seesuay et al. (191)

		Bispecific antibody (FVM09~548 and FVM09~MR72 dual–variable domain immunoglobulin)	20 mg/kg	Pseudo-typed VSV platform	Female BALB/c mice	Specifically and potently neutralize recombinant VSV-EBOV GP in comparison to the parental mAbs FVM09, mAb-548, and MR72 which has poor neutralizing capacity	Wec et al. (189)

6.	Virus replication inhibitors	Okadaic acid (toxin produced by shell fish)	IC_50_ = 130 nM	–	BSR T7/5 cells	Inhibition of protein phosphatases PP1A and PP2A by okadaic acid blocks multiplication of EBOV in target cells	Modrof et al. (263)

		Pyrazinecarboxamide derivative T-705 (favipiravir)	Treatment (300 mg/kg/day)	Used for treating influenza and other segmented viruses	In IFNAR^−/−^ C57BL/6 mice	When treatment initiated 6 days pre-infection or post-infection, it prevented mortality of 100% mice and reduced biochemical correlates of disease	Oestereich et al. (221)
			IC_90_ of 110 µM		Vero E6 cells	Suppression of EBOV replication by 4 log_10_ units	

7.	Nucleotide analog	Adenosine nucleoside analog BCX4430 (interrupt viral RNA synthesis)	16 mg/kg BID dose group	–	Cynomolgus macaque	Significantly prolonged mean time to death	Taylor et al. (203)
			25 mg/kg IM BID (treatment started 30–60 min after inoculation)	–	Rhesus macaque	All animals survived	

		FGI-106	3 mg/kg	Cell-based assays also identified inhibitory activity against divergent virus families	C57BL/6 or BALB/c mice	Single dose of FGI-106, administered 24 h post-infection	Aman et al. (264)

8.	Antivirals	Genistein and tyrphostin AG1478 cocktail	Up to 100 µM	–	HEK 293 cells	Higher concentrations of genistein and lower concentrations of tyrphostin AG1478 has higher inhibition of EBOV	Kolokoltsov et al. (265)

		Carbocyclic 3-deazaadenosine (*S*-Adenosylhomocysteine Hydrolase Inhibitors)	Doses ≥0.7 mg/kg every 8 h	–	Adult BALB/c mice	When treatment initiated at 0 or day 1 post infection, it completely protected animals	Huggins et al. (266)

9.	Oligomer-mediated inhibition	siRNAs targeting the Zaire EBOV RNA polymerase L + VP24 + VP35 in stable nucleic acid-lipid particles (SNALPs)	2 mg/kg total siRNA/dose	–	Chinese rhesus macaques	Macaques given seven treatments with SNALPs were protected after lethal EBOV challenge	Geisbert et al. (249)

		L gene-specific pool of four siRNAs complexed in SNALPs	A single bolus of 0.75 mg/kg siRNA per kilogram of body weight	–	Hartley guinea pigs	One of the 4 siRNAs alone is able to completely protect guinea pigs from a lethal EBOV challenge	Geisbert et al. (267)

		Cell-penetrating peptide conjugated with phosphorodiamidate morpholino oligomers, an uncharged single-stranded DNA analoge; designed to base pair with the translation start site region of VP35	500 µg dose	–	C57Bl/6 mice	Oligomer provided protection to mice when administered before or after an otherwise lethal infection	Enterlein et al. (268)

		TKM-130803	2.24 × 10^9^ RNA copies/ml plasma (0.3 mg/kg)	Single-arm phase II trial, adults with laboratory-confirmed Ebola virus disease (EVD) patients	Human patients	In patients with severe EVD no improvement with treatment	Thi et al. (253)

		miR-607	–	*In silico*	–	Selected mRNA completely blocked all major 4 EBOVs	Golkar et al. (269)

*^a^Dose not mentioned*.

Of the note, a luciferase reporter system (EBOV-like particle/EBOVLP) has been developed. It helps in evaluating the *in vivo* anti-EBOV agents, *viz*., vaccines and drugs without the necessity of biosafety level-4 facilities. The system appears suitable in studying the process of viral entry also (259). The molecular tweezer CLR01 has been recently reported to inhibit EBOV and Zika virus infection. CLR01 interacts with the lipids in the viral envelop but not with the cellular membrane, thereby it is having very less effect on viability of cells (270). This small molecule has earlier been shown to possess antiviral activity against HIV-1 and herpes viruses. Such broad-spectrum antiviral agents need to be further explored to develop an effective drug against EBOV.

Currently, priority is being given toward investigating various proteins in the host system and viral targets (druggable) (271). Further research works need to be strengthened to identify potent viral or host targets that can be exploited to treat EVD or inhibit EBOV. With advances in bioinformatics tools, it is now possible to identify the active sites of the viral targets which can be utilized as a critical step toward designing and discovering anti-EBOV drugs (272). The involvement of computational tools has widened our approach toward designing drugs (target based) widely. Computational approaches can also countervail the endemic burdens in development of drugs traditionally (271, 273). Large libraries can now be effectively screened, ultimately stimulating research activities toward identifying potent anti-EBOV drugs. Therapeutic applications of cytokines, recombinant proteins, RNAi technology/RNA interference, TLRs, avian egg yolk antibodies, plant-based pharmaceuticals, nanomedicines, immunomodulatory agents, probiotics, herbs/plant extracts, and others may be explored appropriately to combat EBOV, as these have been found promising against other viral pathogens (2, 249, 274–282).

## Conclusion and Future Perspectives

The 2014 EBOV outbreak has been marked as the most widespread lethal viral hemorrhagic attack and prompted a hasty leap in the researches for developing effective vaccines and therapies to counter it. In the case of Ebola, deviations in the touchstone drug/vaccine research approaches may be permitted by authorities to an appropriate extent, considering the devastating and alarming pandemic threat from the disease. In recent years, several therapies have emerged to tackle lethal EBOV infections. A plant-derived formulation of humanized mAbs: “ZMapp” has been used to treat some patients. However, the shortage of ZMapp supply warrants the evaluation and development of new mAbs. Various drugs have been repurposed to treat potentially lethal disease like EVD. There is a long list of repurposed compounds that have been evaluated as inhibitors of EBOV, including microtubule inhibitors, estrogen receptor and reuptake modulators, kinase inhibitors, histamine antagonists, and ion channel blockers. In-depth studies are still required to understand the pathogenesis and the role of different EBOV peptides, proteins, and antigens and host–virus interactions in EVD. There is also a need to develop economic and effective antivirals and vaccines against EBOV having approach/utility to any part of the world including resource poor countries.

Although the development of vaccines against EBOV began in 1980, there is still no effective vaccine available to prevent this deadly disease. Hence, the hunt for an effective vaccine is still on. Ebola VLPs play an imperative role in high-throughput screening of anti-EBOV compounds. Because five EBOV species have been reported, a polyvalent vaccine having immunogenic determinants such as GP from each of species would provide broader immunity; indeed, in nonhuman primate experimental studies with a DNA vaccine, this is commonly true. The best first-generation vaccine candidates for EBOV are rVSV and ChAd3, as reflected by their application in providing long duration protection during sporadic outbreaks. Various combinations of antigens from different species of EBOV may be explored to achieve higher protective immune response. The rVSV-based vaccine is being used in Democratic Republic of the Congo. Due to absence of preexisting immunity to VSV, it eliminates several drawbacks and safety concerns associated Ad5-based vaccine. Also, it has show long-term protection in several NHP models, it is an ideal vaccine platform to be used at time of outbreak. Together, the GamEvac-Combi vaccine also seems to be equally promising as it generated immune response in 100% volunteers.

In addition, mAbs with broad cross-reactivity that will neutralize all five species of EBOV are required to be developed and evaluated for prophylactic and therapeutic uses. Furthermore, effective antibodies may be engineered for homogeneity with human antibodies. Many nucleic acid-based modalities like siRNA, miRNA, and PMOs have been tested against EBOV and found functional. In the era of genomics, a computational approach may also be employed to screen large numbers of inhibitory molecules to safeguard human health. Available treatments within the disaster settings; mostly combination of appropriate supportive care and boosting of patient’s immune responses, need to be optimized to ensure minimum research/medical ethics being followed in such settings.

There is always scope for future investigations on the basis of clinical studies that are designed well and statistically supported. Maximum use of supportive therapy (MUST) should be introduced for studying the effects of new therapeutics. The side effects of newer drugs can also be revealed very efficiently by MUST and for this more resources are needed for the Ebola clinics. Though several drugs have been evaluated and vaccines are in development; however, more research is required to develop potent therapeutic and prophylactic agents against EBOV. Apart from these advances, adaptation of appropriate preventive measures and strict biosecurity principles are essential to stop the EBOV outbreaks, limit the spread of virus, and address its public health significance.

## Author Contributions

All the authors substantially contributed to the conception, design, analysis and interpretation of data, checking and approving final version of the manuscript, and agreed to be accountable for its contents. KD, RK, AM, and KK initiated this review compilation. SC, SL, and RK updated various sections. RKS, YM, DK, and MR reviewed virology and biotechnology aspects. RKS, SM, RS, and WC reviewed recent vaccines and therapies. RK designed tables. KK designed the figures. WC, AM, and KD overviewed and edited final.

## Conflict of Interest Statement

All authors declare that there exist no commercial or financial relationships that could in any way lead to a potential conflict of interest.
